# Differential carbon utilization enables co-existence of recently speciated Campylobacteraceae in the cow rumen epithelial microbiome

**DOI:** 10.1038/s41564-022-01300-y

**Published:** 2023-01-12

**Authors:** Cameron R. Strachan, Xiaoqian A. Yu, Viktoria Neubauer, Anna J. Mueller, Martin Wagner, Qendrim Zebeli, Evelyne Selberherr, Martin F. Polz

**Affiliations:** 1 Institute of Food Safety, Food Technology and Veterinary Public Health, Department for Farm Animals and Public Health, University of Veterinary Medicine Vienna, Vienna, Austria; 2 Austrian Competence Centre for Feed and Food Quality, Safety and Innovation FFoQSI GmbH, Tulln, Austria; 3 Division of Microbial Ecology, Centre for Microbiology and Environmental Systems Science, University of Vienna, Vienna, Austria; 4 University of Vienna, Doctoral School in Microbiology and Environmental Science, Vienna, Austria; 5 Institute of Animal Nutrition and Functional Plant Compounds, Department for Farm Animals and Public Health, University of Veterinary Medicine Vienna, Vienna, Austria; 6 Christian Doppler Laboratory for Innovative Gut Health Concepts of Livestock, Vienna, Austria

**Keywords:** Bacterial evolution, Evolutionary ecology

## Abstract

The activities of different microbes in the cow rumen have been shown to modulate the host’s ability to utilize plant biomass, while the host–rumen interface has received little attention. As datasets collected worldwide have pointed to Campylobacteraceae as particularly abundant members of the rumen epithelial microbiome, we targeted this group in a subset of seven cows with meta- and isolate genome analysis. We show that the dominant Campylobacteraceae lineage has recently speciated into two populations that were structured by genome-wide selective sweeps followed by population-specific gene import and recombination. These processes led to differences in gene expression and enzyme domain composition that correspond to the ability to utilize acetate, the main carbon source for the host, at the cost of inhibition by propionate. This trade-off in competitive ability further manifests itself in differential dynamics of the two populations in vivo. By exploring population-level adaptations that otherwise remain cryptic in culture-independent analyses, our results highlight how recent evolutionary dynamics can shape key functional roles in the rumen microbiome.

## Abstract (web summary)

Different populations of Campylobacteraceae co-exist in the cow rumen epithelial microbiome thanks to metabolic trade-offs in utilization of versus inhibition by acetate and propionate, with implications for host carbon uptake.

## Main

Ruminants depend on their microbiome’s remarkable metabolic capacity to digest diverse plant matter, but inefficiencies in feed conversion represent an enormous environmental burden. In addition to demanding over a quarter of Earth’s land and crop mass, current ruminant-based farming practices have a particularly large impact on terrestrial acidification, eutrophication, freshwater usage and methane emissions^1–4^. In fact, many of these consequences go hand in hand and involve interconnected aspects of host and microbial metabolism. For instance, the host’s feed efficiency, the portion of plant biomass that is used for physiological processes such as growth and lactation, is reduced by microbial activities that divert carbon away from the animal^5^. Understanding and potentially limiting these activities while simultaneously promoting those that funnel biomass into metabolites that are readily absorbed by the host is a major goal of rumen microbiome research^6,7^. There have thus been extensive community-level analyses aimed at understanding the microbial sinks and sources of key metabolites^8^. Complementary work has determined how these metabolites influence host nutrition, with a strong focus on acetate, which provides the host with most of the carbon used for de novo lipogenesis, as evidenced by a dose-dependent relationship between acetate and milk fat^9^. Yet, we only have a detailed understanding of the metabolic roles of microbes residing in the lumen, while those attached to the epithelial wall have received comparatively little attention^10–12^. With that said, the microbial groups that are specific to the epithelia have been identified in several 16S ribosomal RNA gene surveys and those that are both dominant and metabolically active have the potential to act as ‘gatekeepers’ of nutrient exchange with the host. One group of microbes that stands out as being particularly abundant and active are previously uncultivated Campylobacteraceae^11,13,14^. We focus on members of this group and ask whether they have distinct abundance distributions and metabolic adaptations with consequences for ruminant nutrition.

To determine how the epithelial Campylobacteriaceae are genotypically structured and to relate this structure to function, we reasoned that a fine-scale genetic and gene flow analysis would allow us to predict niche-specific adaptations and their potential consequences. A recently proposed framework for making such predictions is reverse ecology, which begins by predicting ecologically differentiated populations from genomic information^15^. Such populations are defined as groups of closely related co-occurring bacteria that are characterized by specific adaptations, so that they differ in at least one niche dimension from their sister populations^16^. There are two principal modes by which adaptations can spread through populations and differentiate them^17^: The first mode is via gene-specific sweeps where a novel adaptive gene or allele spreads within a bacterial population that represents a cohesive gene flow unit due to higher recombination within than between other such units^18,19^. The second mode is via genome-wide sweeps where the entire genome hitchhikes with an adaptive gene or allele resulting in a highly clonal population structure^20^. Detecting populations via these evolutionary modes is therefore useful for resolving units with niche-differentiating adaptations, which, as the final step in the reverse ecology framework, can be subsequently quantified and linked to specific functional roles and dynamics within the microbiome^21^. To provide an example, a recent application of reverse ecology was able to use gene flow information to predict populations of *Ruminococcus gnavus*, show that they were subject to specific gene-specific sweeps and then use this information to link these populations to healthy and disease states in the human microbiome^21^.

Following the above logic, we explored the population structure of a lineage of Campylobacteraceae found to be most abundant and active in the cow rumen epithelia microbiome based on the analysis of metagenome-assembled genomes (MAGs). We then carried out a reverse ecology approach enabled by isolate genomes that split this Campylobacteraceae lineage into two closely related populations that bear the hallmarks of genome-wide selective sweeps indicating ongoing speciation. Searching the genomes for population-specific, potentially adaptive features revealed extensive similarity in terms of genomic synteny and core metabolic potential, while the few genetic differences present were suggestive of differential colonization strategies. However, no spatial pattern in colonization was discernible, suggesting the populations co-occur, leading us to further explore whether gene expression differed among the Campylobacteraceae populations. We leveraged in vivo transcriptomes, which predicted modifications in the regulation of acetate utilization and led us to notice a duplication event within the underlying genes. Growth and fitness assays with representative strains then exposed a trade-off in competitive ability where one population can grow better on acetate but is inhibited by propionate, while the other population showed no detectable growth advantage or disadvantage with either substrate. Being diet-dependent cornerstones of ruminant nutrition, acetate and propionate are commonly measured in feeding trials, which allowed us to detect correlations with individual populations that are consistent with the observed trade-off. Taken together, the results highlight how metabolic differences resulting from micro-evolutionary processes structuring populations may impact the availability of short-chain fatty acids (SCFAs) to the animal.

## Integrating datasets of different origin and resolution

As several 16S rRNA amplicon surveys of the rumen epithelial microbiome reported a particularly dominant operational taxonomic unit belonging to the family Campylobacteraceae^22,23^, which is only detected at very low abundances in the rumen content^23,24^, we aimed to explore the diversity represented by this operational taxonomic unit by using approaches of increasing resolution. As described in the following sections, we first demarcate host-attached bacteria into 16S rRNA gene amplicon sequence variants (ASVs), then MAGs and finally fine-scale clusters of isolate genomes. Doing so provided reference genomes that enabled us to further map additional amplicon and metatranscriptomic data, some of which were previously collected from the same animals, while the rest were collected from globally distributed animals and obtained via publicly available databases. The sources and details of all the data we integrate in this study are listed in Supplementary Table 1.

## Ubiquity and activity of rumen epithelial Campylobacteraceae

**Fig. 1:**
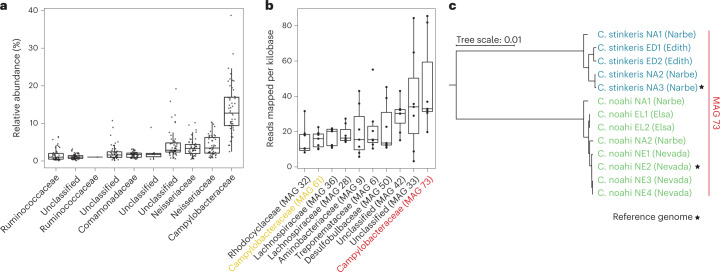
Abundance and microdiversity of Campylobacteraceae from the rumen epithelia. **a**, Relative abundance of the top ten 16S rRNA gene ASVs based on the merging and re-analysis of two recent studies^22,23^. The points represent samples where no specific feed additives were applied and that were taken from eight cows at different times (*n* = 48). **b**, Metagenomes were sequenced for seven different cows and assembled into MAGs (*n* = 7). The reads mapped per kilobase are shown for the ten most abundant MAGs with a completeness of over 50% and under 10% contamination. Box plots show the median with hinges that correspond to the 25th and 75th percentiles. The whiskers extend from the hinge to the largest and smallest value no further than 1.5 multiplied by the inter-quartile range. Individual data points are overlaid. **c**, Phylogenetic tree reconstructed with 13 isolate genomes that correspond to MAG 73 in **b** (Extended Data Fig. 4). Whole-genome alignments at the nucleotide level spanned 1.3 Mb. The two closely related populations are named ‘*Ca*. C. stinkeris’ and ‘*Ca*. C. noahi’ for convenience. The names given in brackets are the names of the animals from which we obtained the isolate genome. The genomes of the two marked isolates were closed and are used as reference genomes in subsequent analysis.

**Extended Data Fig. 1:**
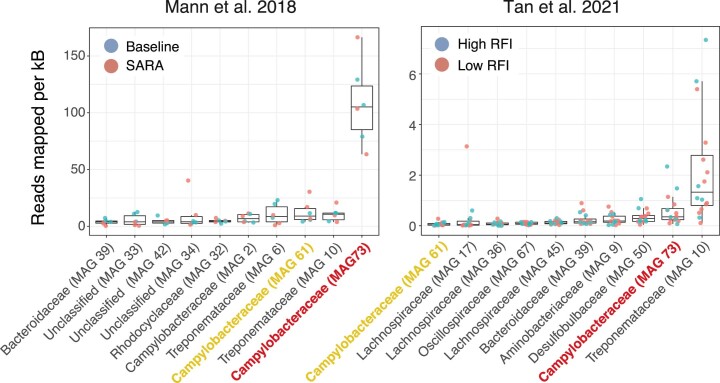
Relative activity of MAGs in different metatranscriptomic datasets. Data from two transcriptomic studies^13,83^ were mapped against the rumen epithelial MAGs. Reads mapping to predicted ORFs in the MAGs were counted and the reads mapped per kilobase (kB) were plotted for each treatment group. Mann *et al*.^13^ assessed the impact of sub-acute ruminal acidosis (SARA) on dairy cattle in Austria (n = 6), while Tan *et al*.^83^ compared beef cattle with low and high relative feed intake (RFI) in Canada (n = 16). Box plots show the median with hinges that correspond to the 25th and 75th percentiles. The whiskers extend from the hinge to the largest and smallest value no further than 1.5 multiplied by the inter-quartile range. Individual data points are overlaid.

By first merging amplicon sequence data from two recent studies^22,23^, we saw that a single ASV belonging to the Campylobacteraceae was indeed most abundant, but highly variable across individual cows, with a median relative abundance and coefficient of variance of 15% and 50%, respectively (Fig. 1a and Supplementary Table 1). This ASV was detected in all 48 epithelial samples we analysed, which were taken from animals that took part in multiple feeding trials (eight animals in total; for details, see Methods). To further characterize the diversity and abundance of these Campylobacteraceae, we sequenced metagenomes from seven of the same animals and calculated the coverage of MAGs (Fig. 1b). Consistent with the amplicon sequence data, one of the MAGs classified as Campylobacteraceae (MAG 73) was overall most abundant but varied substantially between the different animals (coefficient of variance of 58%). A second Campylobacteraceae MAG (MAG 61) was among the most abundant MAGs, but recruited nearly threefold fewer reads than MAG 73. MAG 73 was also found to be highly active in terms of total gene expression and recruited approximately tenfold more reads than MAG 61 in two metatranscriptomic datasets (Extended Data Fig. 1). We then aimed to isolate strains representing the two MAGs, which could be used to assess both their relatedness to each other and position within the Campylobacteraceae phylogeny.

## Dominant Campylobacteraceae share a 16S rRNA but are distantly related

**Extended Data Fig. 2:**
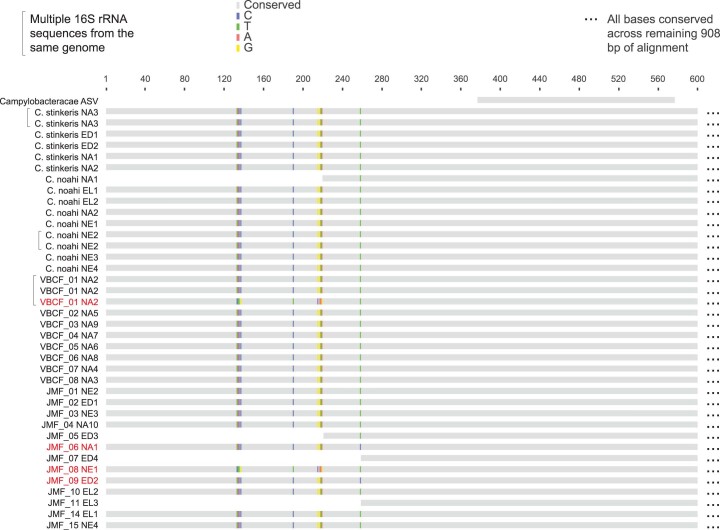
The 16 S rRNA gene is conserved across the genomes sampled. The 16 S rRNA gene was extracted from all genomes and aligned. The topmost sequence is the ASV found to be most abundant in our reanalysis of two rumen epithelial amplicon studies^22,23^. Only the first 600 of 1508 positions are shown, as there was no variation observed across the rest of the alignment. On the left had side, sequences that were extracted from the same genome are shown (indicated with brackets). Two variants were detected in 4 genomes and are indicated by the genome names in red. The most divergent of the two variants (9 bp changes) is represented as one of the copies of the rRNA gene within the closed reference genome VBCF_01 NA2. A single base pair change defines the remaining variant detected in genomes JMF_06 NA1 and JMF_09 ED2.

**Extended Data Fig. 3:**
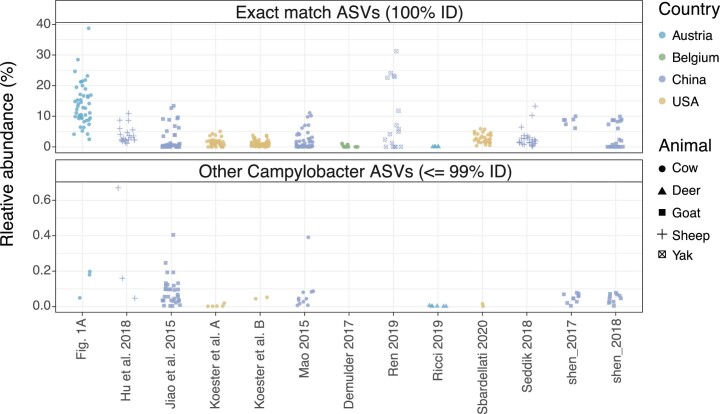
ASVs matching the genomes collected in this study across different animal hosts in various countries. Relative abundance of ASVs that either perfectly matched the 16 S rRNA of the Campylobacteraceae genomes obtained in this study (100% identity) and other ASVs classified as Campylobacteraceae but with 99% identity or less. Amplicon data was analysed from 12 studies that sampled the rumen epithelia and were previously analysed together by Anderson *et al*.^11^. The same data from the amplicon reanalysis in Fig. 1a is also shown for comparison.

**Extended Data Fig. 4:**
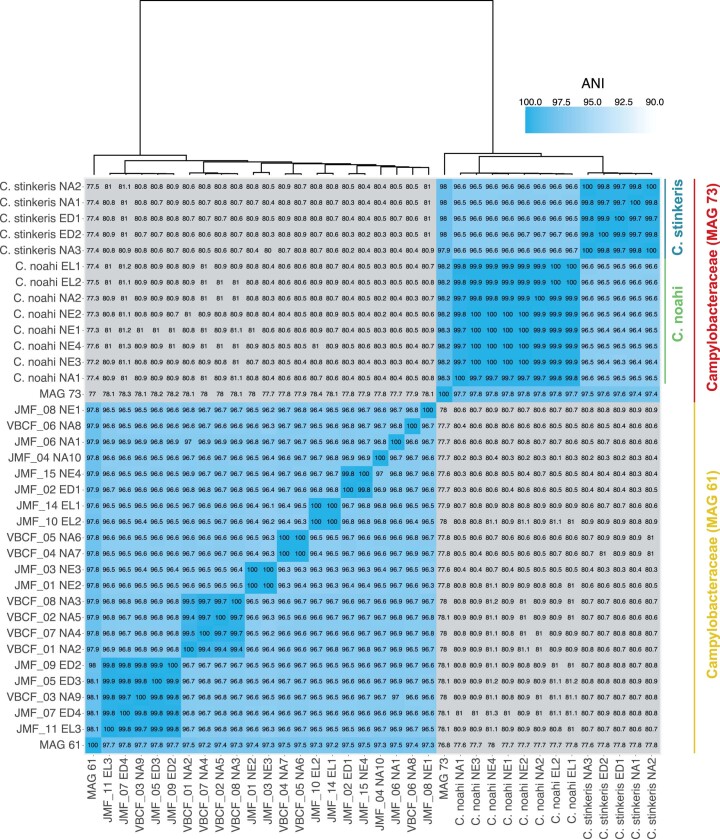
Pairwise ANI and clustering of genomes with MAGs suggests population structure. Genomes were compared pairwise using FastANI^72^ and the resulting ANI values were hierarchically clustered using a complete linkage algorithm. The resulting clusters are represented by a dendrogram on the top and are named, as referred to throughout the paper, on the side of the heatmap. Values representing comparisons of genomes from the two major clusters (containing MAG 61 and 73) are coloured in grey.

**Extended Data Fig. 5:**
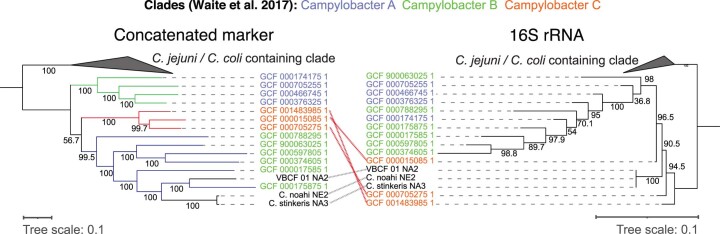
Phylogenetic structure of novel Campylobacteraceae sp. and genes involved in horizontal gene transfer. Face-to-face comparison of a concatenated marker and 16 S rRNA phylogeny coloured based on a recently proposed taxonomy by Waite *et al*.^25^. The same genomes analysed by Waite *et al*.^25^ were used here and combined with the 3 closed, reference genomes obtained in this study. The concatenated marker alignment was made using the GTDB toolkit^84^. The full-length 16 S rRNAs were extracted from the same genome used in the concatenated marker tree and are labelled by their accession in RefSeq.

Metatranscriptomics-informed cultivation recovered isolates that represented both MAGs, but that cannot be resolved by the 16S rRNA gene. Namely, we noticed a highly expressed nitrate reductase, which could be assigned to the family Campylobacteraceae^13^. We therefore supplemented anaerobic medium with nitrate as a terminal electron acceptor, leading to the isolation of 34 strains with 16S rRNA gene sequences that are either identical to each other (31 strains) or explained by operon variation within genomes (Extended Data Fig. 2). All these 16S rRNA sequences encompassed the abundant Campylobacteraceae ASV. Importantly, other ASVs that exactly match the strains’ 16S rRNA are highly abundant in rumen epithelial samples taken from different ruminant species throughout the world, whereas related sequences are detected at much lower abundances (Extended Data Fig. 3 and Supplementary Table 1). Pairwise ANI comparisons showed that the genomes from these strains cluster tightly with the two MAGs and that these two groups share only 80% ANI on average, similar to the divergence between the MAGs (Extended Data Fig. 4). Considering the discrepancy between genome and 16S rRNA sequence identity (80% versus 100%), we wanted to assess how the sequences group with those recently classified. We thus compared both a genome-wide and a 16S rRNA phylogeny that included representative genomes from each cluster and those from a recent re-classification of the Epsilonproteobacteria (Extended Data Fig. 5)^25^. This revealed that the rumen epithelial genomes group with the *Campylobacter* B clade, as per Waite et al.^25^, and are only distantly related to the pathogens *Campylobacter jejuni* and *Campylobacter coli*. The discrepancies between the two phylogenies also suggested horizontal transfer of the 16S rRNA gene (Extended Data Fig. 5), which, as a consequence, does not resolve the two major clusters represented by the MAGs (Extended Data Figs. 2 and 4). As MAGs are co-assemblies of related bacteria that effectively collapse the sampled genome diversity, we refer to genomes that cluster tightly with one of the two Campylobacteraceae MAGs as belonging within that MAG. We next asked whether these MAGs represent two cohesive populations or whether further population-level differentiation exists within a MAG.

## Fine-scale genomic structure within the MAGs

Phylogenetic analysis of the isolates’ genomes suggested further fine-scale differentiation of the MAGs. Within the more abundant and active MAG 73, two well-sampled, microdiverse clusters consisting of five and eight genomes from multiple animals were observed (Fig. 1c). These clusters shared 96.6% ANI and were considered candidate populations because they fall below what is often considered species-level differentiation (<95% ANI) and the within-population divergence is very low (<0.4%) (‘*Ca*. C. stinkeris’ and ‘*Ca*. C. noahi’ in Extended Data Fig. 4)^26^. Although some strains within MAG 61 are also closely related, the majority of the sampled isolates consist of single or pairs of genomes that are approximately equidistant from each other (Extended Data Fig. 4). In either case, comparing isolate genomes with the MAGs suggested that there is additional population structure, based on sampling multiple genomes from microdiverse clusters within each group, but that this structure was less diverse and better sampled within MAG 73. Considering this and that MAG 73 is the dominant group in vivo, we carried out a detailed analysis with the genomes from the 13 sampled members therein to assess their mode of diversification and differentiating features by comparative genomics.

## Campylobacteraceae populations have been shaped by genome-wide sweeps

**Extended Data Fig. 6:**
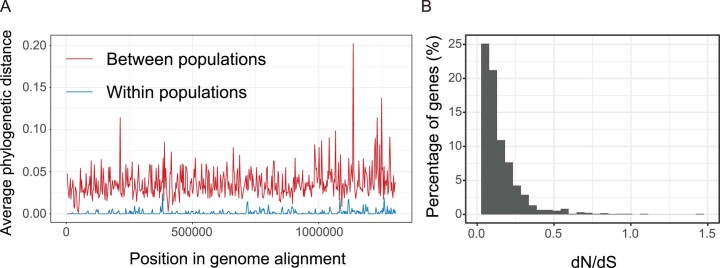
Low within-population genome-wide diversity is likely the result of selection. **a)** The average phylogenetic distance within and between populations along an alignment of all ‘*Ca*. C. stinkeris’ and ‘*Ca*. C. noahi’ genomes. The whole-genome alignment was divided into 2.5 kB windows and a phylogenetic tree was calculated for each alignment window. **b)** The distribution of dN/dS ratios for gene alignments between populations.

We hypothesized the two microdiverse clusters within MAG 73 to represent populations with distinct ecological niches that have been shaped by selection in the process of speciation. This hypothesis was made in consideration of the phylogenetic structure and mutational signatures indicating selection. First, the two genome clusters consist of very closely related genomes, which share a recent common ancestor as indicated by their nucleotide divergence of <0.004 (Fig. 1c). These genome clusters are connected to each other by long branches that are relatively stable across the genome and correspond to a sequence divergence of ~0.034 (Fig. 1c and Extended Data Fig. 6a). Such genetic structure is consistent with genome-wide selective sweeps where selection favours a genome carrying an adaptive mutation over its immediate kin allowing it to outcompete other genomes occupying the same niche over time^20,27^. Eventually, this process leads to a highly clonal population structure, evident as the brush-like structure observed in the phylogeny. Drift as an alternative explanation for the origin of these genome clusters is highly unlikely because diversity within clusters should not differ much from that between clusters due to the accumulation of neutral mutations^28^. Moreover, simulations have shown that clusters, if they form by drift, are short lived because recombination rapidly homogenizes them with the parent population^17^. Finally, the mutations that have accumulated since the divergence of the two populations indicate strong purifying selection (Extended Data Fig. 6b)^29,30^.

As a corollary, for sister populations differentiated by the process outlined above to co-occur in samples, they must have sufficiently reduced niche overlap, either manifesting as differential spatial associations or trade-offs in competitive ability that allow overlapping co-existence^31–34^. We reasoned that the two abundant populations from MAG 73 (‘*Ca*. C. stinkeris’ and ‘*Ca*. C. noahi’ in Fig. 1c) represent a tractable model to test these theoretical expectations and applied a reverse ecology approach^15^. By leveraging genomes, metagenomes and metatranscriptomics, we aimed to test the hypothesized population structure and further predict differentiating features that could be probed experimentally.

## Population-level differentiation involves gene import and gene-specific sweeps

**Fig. 2:**
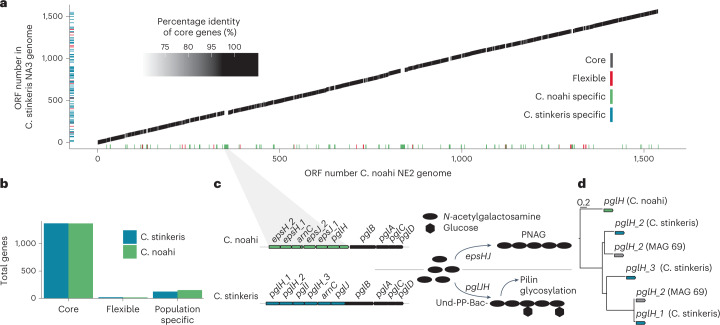
The two dominant Campylobacteraceae populations have highly similar gene content but divergent pgl operons. **a**, Percentage identity of shared genes between ‘*Ca*. C. stinkeris’ NA3 and ‘*Ca*. C. noahi’ NE2 numbered by their order in the reference genomes starting with the same gene. The remaining 11 genomes were further leveraged to label genes as core, flexible or population specific. Core genes are those found in all 13 genomes. Lines plotted directly on the axes are flexible genes, defined as genes missing from two or more of any of the ‘*Ca*. C. stinkeris’ or ‘*Ca*. C. noahi’ genomes. Those flexible genes that are unique to and found in all genomes of one of the populations are marked as ‘population specific’. **b**, Summary of the number of core, flexible and population-specific genes. **c**, Cluster of annotated ‘*Ca*. C. noahi’ genes that are adjacent to the pgl operon and include four *eps* (exopolysaccharide) genes. The predicted role of these genes in producing poly-*N*-acetylgalactoseamine is shown to the right. Below is the corresponding region in ‘*Ca*. C. stinkeris’, which in contrast has variants of the *pglH* gene. This gene is predicted to code for an enzyme involved in polymerizing *N*-acetylgalactoseamine for protein glycosylation. **d**, A protein tree constructed using amino acid sequences for each of the *pglH* genes. Homologous pglH protein sequences were also added from an Elusimicrobiota MAG (MAG 69) sequenced in this study (Supplementary Table 3). The average pairwise amino acid identity is 40%.

**Extended Data Fig. 7:**
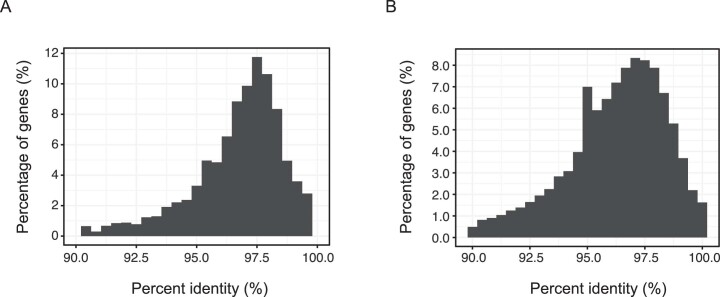
Between population diversity is similar in genomic and metagenomic data. **a)** Gene identity distribution of reciprocal blast hits between populations at the nucleotide level. **b)** Metagenomic reads that competitively mapped to one population were cross-aligned to the other sister population using blast. The distribution of the identity of cross-aligned reads is shown.

To explore potential mechanisms of differentiation between ‘*Ca*. C. stinkeris’ and ‘*Ca*. C. noahi’, we compared all isolate genomes from the two populations in terms of shared gene content. As we were only able to obtain a small set of isolates for each population (five and eight for ‘*Ca*. C. stinkeris’ and ‘*Ca*. C. noahi’, respectively), we first assessed whether additional, closely related populations might be present in samples. We did this by comparing inter-population diversity in the genomic and metagenomic data, as additional populations present in the metagenomic data would be expected to cause a shift in the sequence identity distribution, but we found no evidence for populations that are missing in our genome collection (Extended Data Fig. 7). We then aligned open reading frames (ORFs) from the genomes at the nucleotide level and detected ‘core’, ‘flexible’ and ‘population specific’ genes. By plotting the percentage identity of genes shared between the complete reference genomes obtained for ‘*Ca*. C. stinkeris’ and ‘*Ca*. C. noahi’, we observed a large syntenic core genome that accounted for approximately 90% of the total gene content (Fig. 2a). The majority of the flexible genome fraction was specific for one of the populations (Fig. 2b), but these were largely annotated as hypothetical (77%, Supplementary Table 2). Among the remaining 65 annotations were no apparent core metabolic pathways (Supplementary Table 2). With that said, five of the genes in ‘*Ca*. C. noahi’ were annotated as encoding glycosyl transferases and fell into a cluster adjacent to the pgl (protein glycosylation) operon (Fig. 2c)^35^. These genes included four *eps* (exopolysaccharide) genes that encode enzymes for utilizing the same substrates as the pgl operon but to synthesize poly-*N*-acetylgalactosamine, a biofilm component involved in bacterial adhesion^36^. Within the corresponding region of ‘*Ca*. C. stinkeris’, however, there are several variants of *pglH*, a key gene underlying protein glycosylation (Fig. 2c)^37^. The corresponding pglH proteins are highly divergent and often cluster tightly with homologues found in distantly related bacteria from the rumen epithelia (Fig. 2d and Supplementary Table 3). This suggests that these genes were introduced by horizontal gene transfer, consistent with previous analyses showing that multicopy genes most often arise by transfer rather than duplication and divergence^38,39^. Together the differences in gene content in ‘*Ca*. C. noahi’ and ‘*Ca*. C. stinkeris’ suggest adaptions for colonization strategies that differ in the specifics of biofilm formation.

**Fig. 3:**
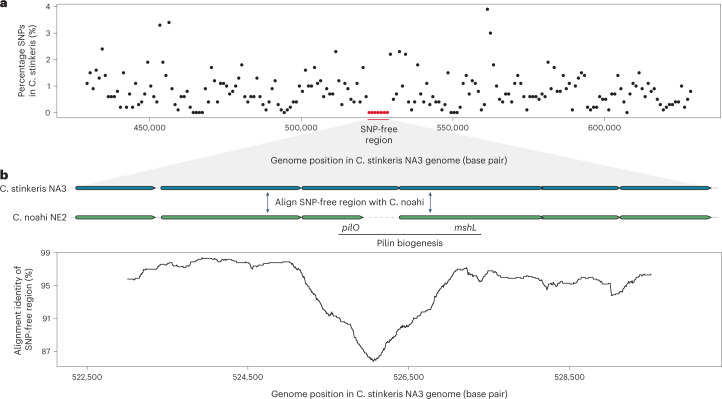
Gene-specific sweeps involving pilin biogenesis genes in ‘*Ca*. C. stinkeris’. **a**, SNPs were called in ‘*Ca*. C. stinkeris’ after competitively mapping of metagenomic reads to the reference genomes from both populations. The percentage of detected SNPs was calculated in 1,000 bp windows and plotted. No SNPs were observed in a region spanning over 7 kB, the largest SNP-free region. **b**, The region identified in **a** was aligned between ‘*Ca*. C. stinkeris’ NA2 and ‘*Ca*. C. noahi’ NE3. The region included two genes annotated to be involved in pilin biogenesis, *pilO* and *mshL*. The *pilO* gene has been interrupted by a stop codon. Alignment identity of the region was calculated in 1,000 bp windows and plotted as a moving average.

As adaptive alleles have also been shown to spread within populations by homologous recombination (gene-specific sweeps), leading to reduced nucleotide diversity compared with the rest of the genome within the affected loci, we analysed the intrapopulation single-nucleotide polymorphism (SNP) distribution across genomes of both populations^18,21^. To best capture genome-wide diversity, SNPs were called by competitively mapping metagenomic reads to the reference genomes from each population, revealing a large (>7 kB) SNP-free region in ‘*Ca*. C. stinkeris’ (Fig. 3a). No such region was detected in ‘*Ca*. C. noahi’. Aligning the SNP-free region from ‘*Ca*. C. stinkeris’ to ‘*Ca*. C. noahi’ showed a sharp decrease in alignment identity that falls within two genes annotated as being involved in pilin glycosylation and biogenesis (Fig. 3b)^18,32,40^. This pattern is consistent with the recent, that is, after the last genome-wide sweep, acquisition of this SNP-free region from a distant but unknown source followed by it spreading via a gene-specific sweep within ‘*Ca*. C. stinkeris’. Hence, in further agreement with the prominent role of recombination in other Campylobacteraceae, which have been shown to readily import genes from other species^41–43^, we show that, in addition to genome-wide sweeps, gene-specific sweeps are involved in differentiating the populations. Accordingly, the two pilin biogenesis genes are probably under differential selection and, in line with this notion, one of the two genes, *pilO*, has become truncated in ‘*Ca*. C. noahi’ (Fig. 3b). Altogether, population-specific gene content and signatures of selection support the proposed population structure and predicted modifications in pili and biofilm formation, which led us to hypothesize divergent colonization strategies and thereby spatial separation on the epithelial wall.

## No evidence for spatial separation on the rumen wall

**Extended Data Fig. 8:**
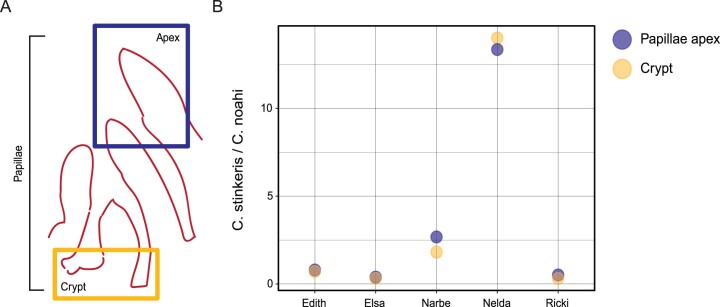
The ratio of ‘*Ca*. C. stinkeris’ to ‘*Ca*. C.noahi’ at two different locations along the papillae. **a**) Cartoon based on a representative photomicrograph^85^ of rumen papillae to show the two sections that were dissected from papillae biopsies. From these, DNA was extracted, and the populations were measured using dPCR. **b**) The ratios of the populations are plotted as we hypothesized divergent distributions of the two populations across the papillae. Individual data points are shown and there was no significant difference between the apex and crypt samples (two-sample t-test, *P* = 0.98). The names on the x-axis are the names of the different animals (n = 5) from which we obtained the papillae samples.

To test for potential spatial separation of the two populations, we used digital polymerase chain reaction (dPCR) to measure their distribution across papillae samples. Papillae are projections that cover the rumen wall to increase surface area for the absorption of SCFAs^44^. We dissected the apex and the crypts of the papillae (Extended Data Fig. 8a), representing the furthest and nearest tissue connected to the epithelial wall, respectively, which would be expected to harbour different ratios of the populations when assuming a gradient of opposite relative abundances across the papillae. However, the ratio of the two populations appeared similar in the two dissected samples, despite large differences between animals (Extended Data Fig. 8b). Of course, it is still possible that smaller scale spatial associations exist, beyond the resolution of our dissection strategy, but considering that we do not detect differences at opposite ends of single papillae and that the outer layer of the papillae is gradually sloughed off in vivo, interaction between the populations seems likely. We therefore reasoned that some additional mechanism might be present to minimize competition. Such a mechanism would need to support co-existence of the two populations by preventing overlap in their growth dynamics via differentiation in some metabolic niche dimension.

## Differential gene expression points to differences in acetate utilization

**Fig. 4:**
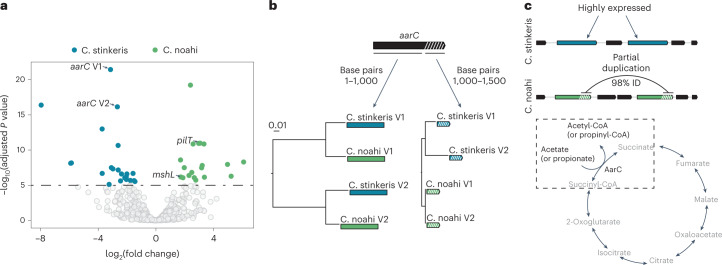
In vivo expression and analysis of evolution of an acetate acetyl-CoA transferase (AarC) that differentiates ‘*Ca*. C. stinkeris’ and ‘*Ca*. C. noahi’. **a**, Volcano plot showing the range of fold changes and corresponding adjusted *P* values from differential expression analysis using DESeq2 comparing the two populations with the metatranscriptomic data from Mann et al.^13^ (Supplementary Table 1). The horizontal line indicates significant genes with a adjusted *P*-value cut-off of 0.00001 (*n* = 6, Benjamini–Hochberg method for adjusted *P* values, two-sided; Supplementary Table 4). The two variants of *aarC* are marked. *mshL* is also marked as it corroborates the analysis in Fig. 3. **b**, Phylogenetic trees at the gene-level for different regions of the two variants of *aarC*. The two trees were reconstructed using different segments of the four *aarC* variants (two per genome), either the first 1,000 bp or the last 500 bp. **c**, Graphical summary of results and hypothesis from the analysis in **a** and **b**. The gene diagrams show that two different variants of the *aarC* gene are highly expressed in ‘*Ca*. C. stinkeris’ relative to ‘*Ca*. C. noahi’ wherein a segment of the *aarC* genes has been homogenized. AarC is CoA transferase predicted to assimilate acetate via the tricarboxylic acid cycle, but has also been shown to have activity on propionate, leading to propionyl-CoA^48^.

As comparative genomics suggested that metabolic genes are shared among the two populations, we hypothesized that their individual metabolisms might be rewired in ways that are not easily predicted by annotation^45^. We therefore aimed at comparing ‘*Ca*. C. stinkeris’ and ‘*Ca*. C. noahi’ on the regulatory level. Leveraging in vivo transcriptomes, we carried out competitive mapping between the representative genomes of each population. Among the most differentially expressed genes in ‘*Ca*. C. noahi’ was the pilin biogenesis gene, *mshL*, corroborating that this gene, hypothesized to be under differential selection in the analysis of gene-specific sweeps (Fig. 3 and Supplementary Table 4), is involved in population-specific ecology in vivo. Additionally, two of the most highly differentially expressed loci in ‘*Ca*. C. stinkeris’ implicated two variants of the metabolic gene, *aarC* (V1 and V2 in Fig. 4a), that code for an enzyme that assimilates acetate via the tricarboxylic acid cycle^46,47^. On average, the variants are quite divergent from each other when compared across the two populations, with the more divergent of the two variants falling two standard deviations outside of the mean gene identity (90.9% versus 96.6%; Supplementary Table 5). By comparing the variants in detail, we noticed that a segment coding for the C-terminal end (the last 500 of the total 1,500 base pairs) was highly similar in the two copies of the ‘*Ca*. C. noahi’ *aarC*s. Gene trees including the variants from both populations were therefore constructed using the two segments of the gene, the larger of which implied an ancestral duplication of *aarC*. Yet, the shorter segment appears to have been recently transferred within the ‘*Ca*. C. noahi’ (Fig. 4b). Overall, these observations suggest that fine tuning at both the regulatory and allelic level has occurred since population divergence. Such fine tuning may be an indicator of metabolic differentiation, as the most related AarC is a CoA transferase acting primarily on acetate but also to a degree on propionate (Fig. 4c)^48^. As these two metabolites are the most abundant products of rumen fermentation and main energy substrates for the host^7^, we wanted to test whether the two populations interact differently with acetate and propionate.

## Growth dynamics in vitro and in vivo reveal a trade-off in competitive ability

**Fig. 5:**
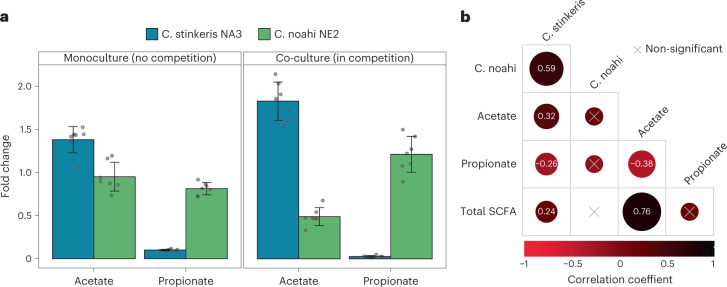
An apparent trade-off in acetate utilization and propionate resistance in vitro and in vivo. **a**, Growth experiments assessing the effect of adding 5 mM acetate or propionate on biomass accumulation by population-specific dPCR. Growth differences were assessed on agar since the strains did not grow in liquid (Methods). Fold change is shown relative to control (no SCFA added). Experiments with individual strains (left, monoculture) and in competition (right, co-culture) were performed. Bar plots show the mean ± standard deviation (*n* = 7). Individual fold changes are also overlaid. **b**, Correlations (Pearson) between population-specific dPCR data and rumen SCFA concentrations (*n* = 94) (ref. 23). Coefficients are indicated for significant correlation tests (*P* < 0.05, two-tailed; for details, see Methods). Data were obtained from samples taken during a previously performed feeding trial^23^ that used the same cows (*n* = 8) from which the strains in this study were cultivated.

Growth assays with the representative strains showed that ‘*Ca*. C. stinkeris’ accumulated more biomass when provided with acetate as the main carbon source but appeared to be inhibited by propionate (Fig. 5a). In contrast, ‘*Ca*. C. noahi’ did not accumulate any measurable change in biomass with either of the SCFAs (Fig. 5a). A relative fitness advantage afforded by acetate and propionate to ‘*Ca*. C. stinkeris’ and ‘*Ca*. C. noahi’, respectively, was also observed when the strains were competed against each other. Specifically, acetate led to ‘*Ca*. C. stinkeris’ outcompeting ‘*Ca*. C. noahi’ by over threefold, while the same concentration of propionate led to complete dominance by ‘*Ca*. C. noahi’ (Fig. 5a). We then tested whether these effects were supported by in vivo relative abundances by analysing population dynamics in a recent feeding trial during which a time course of epithelial samples were collected and SCFA concentrations were measured in the rumen^23^. We used the population-specific dPCR assay and correlated ‘*Ca*. C. stinkeris’ and ‘*Ca*. C. noahi’ abundance in the epithelial samples with SCFAs. In line with their relative fitness observed in vitro, the ‘*Ca*. C. stinkeris’ population correlated positively with acetate and negatively with propionate, while ‘*Ca*. C. noahi’ showed no notable correlation with either fatty acid (Fig. 5b). Considering that the two populations are otherwise predicted to utilize the same types of electron acceptors and donors based on the overlap in metabolic gene annotations, our data show that the two populations possess unique fine-scale adaptations, including changes in regulation and structure of the AarC enzyme. These adaptations appear to have lessened the ability of ‘*Ca*. C. noahi’ to utilize acetate but made it more resistant to propionate. Altogether, we provide evidence for a trade-off in competitive-ability-based acetate utilization and propionate resistance that in turn may lead to different dynamics depending on the flux of acetate and propionate^49^.

## Discussion

Microbes attached to the rumen epithelia have received little attention compared with those in the lumen, but by forming a biofilm at the host–rumen interface, they may have a disproportionate influence on the flux of metabolites entering the host. By characterizing one of the most abundant epithelia-attached microbes and following a reverse ecology framework^15,21^, we shed light on a sink for acetate, the main fermentative end product of the rumen microbiota and essential carbon source for the animal^9^. The logic of the reverse ecology approach is that it starts with detection of genomic units, referred to here as populations, that have been optimized by selection to be ecologically differentiated. Hence, any population specific differences may provide information on the forces that have driven population differentiation or further optimized them. Accordingly, we were able to predict sister populations from closely related isolate genomes and show that a key differentiating trait is acetate utilization. This difference was not predictable by annotation alone, as both populations have the genomic potential to use the same electron donors and acceptors. If this pattern of fine-scale metabolic differentiation observed in SCFA utilization is more general and involves other metabolic traits, then current cultivation-independent approaches, such as the analysis of MAGs, may be insufficient to accurately assign important functional roles or specific cross-feeding interactions within microbiomes.

Acyl-CoA transferases like the AarC enzyme appear to be more generally involved in differentiating important metabolic roles of closely related bacteria in the microbiome. A recent comprehensive study of Bacteroidetes in the human microbiome uncovered a trade-off similar to the one described here that involves acyl-CoA transferases acting on butyrate^50^. This has the exciting implication that there may be generalizable trade-offs involving acyl-CoA transferases that act on SCFAs. As SCFAs have been found to be involved in several microbiome–host interactions^51–53^, understanding the specifics of these trade-offs will provide a more mechanistic framework for designing microbiome-targeted interventions. In the rumen, these could aim to modulate the ratio of acetate to propionate without requiring dietary modifications. With that said, there are many other functional roles carried out by the microbiome, in addition to those involving SCFAs, that could influence nutritional outcomes and be highly differentiated on a population level. An interesting example is reactive oxygen species removal, which was discussed in a recent study that implicated the rumen epithelial Campylobacteraceae in feed efficiency^53^.

The clonal structure of the populations observed here suggests that they are the result of recurrent genome-wide selective sweeps, that is, they are on a speciation trajectory. This view is supported by the relatively high divergence between and low diversity within the populations. Practically, this structure provides a convenient means to define populations, fundamental units that have been differently optimized by selection and therefore can be hypothesized to have differential associations or dynamics. Pinpointing the underlying adaptions that initiated the differentiation, however, is complicated by the fact that genome-wide sweeps purge diversity in the population, a form of hitchhiking involving the entire genome^54,55^. In fact, genome-wide sweeps are expected if selection is strong relative to recombination^56^. Conversely, if selection is relatively weak in recombining populations, gene-specific sweeps may happen and are evident as reduced diversity within a locus in a population. Indeed, our analysis provides evidence that the two populations studied here have also been optimized by gene-specific sweeps relatively recently, that is, after the last genome-wide sweep. These gene-specific sweeps have probably involved several differentiating loci, including the C-terminal end of the two variants of *aarC* in ‘*Ca*. C. noahi’. This may indicate that shifting feeding strategies, such as increased reliance on grain, that elevate SCFA production in the gut have rapid selective feedback on the evolution of microbial populations. Although it is impossible to know the triggers of the initial population differentiation, we speculate that it occurred during the agricultural revolution with dramatic changes in feeding practices as cattle were more intensively reared. Supporting this idea is the fact that ‘*Ca*. C. stinkeris’ and ‘*Ca*. C. noahi’ are more closely related than clades 1 and 2 *Campylobacter coli*, which were shown to have diverged from each other approximately 1,000 years ago^57^. It is therefore possible that shifts in feed composition initiated the process of divergence, which is still ongoing today as cattle production is increasingly industrialized.

There is an urgent need to reduce the impact of intensive ruminant-based agriculture. While several inefficiencies in these systems will need to be tackled simultaneously, a promising means of intervention is the rumen microbiome^5,6,8^. Our work demonstrates that microbial adaptation to shifts in feed can be rapid and involve changes in the utilization of key carbon sources at the population level. These findings may help us to understand and influence the flux of nutrients into the host with the goal of decreasing the extent to which food crops are required by high-performance animals.

## Methods

### Amplicon data re-analysis

All publicly available amplicon datasets were downloaded using fastq-dump (v.2.11.0. Supplementary Table 1). Rumen epithelial amplicon data were first downloaded from two studies^22,23^ that amplified the same variable region of the 16S rRNA gene (V3–V5), and the data were reprocessed using the qiime2 environment (v. 2021.4.0) (ref. 58). The forward reads from both datasets were denoised by implementing dada2 with trimming from positions 25 to 225 and 42 to 242 for the Neubauer et al.^23^ and Wetzels et al.^22^ data, respectively. The resulting count tables were merged by shared, identical ASV sequences and classified using the rRDP package (v. 1.20.0) (ref. 59) in R (v. 3.6.3) (ref. 59). The relative abundances for each ASV in each sample were also calculated in R (v. 3.6.3), where the top ten most abundant ASVs based on their median relative abundance across all samples were plotted in Fig. 1a.

For the rumen amplicon data shown in Extended Data Fig. 3, the same approach as above was followed, with the exception that the dataset from each study^11^ was processed individually with trimming from positions 20 to 220. The resulting ASVs were compared with the 16S rRNA gene sequences from the genomes obtained in this study (see ‘Genome sequencing and diversity analysis’ below). The relative abundance of ASVs with 100% sequence identity across their entire sequence length was plotted as ‘exact matches’. For other ASVs that were classified as Campylobacteraceae with 99% identity or less sequence identity, the relative abundance was plotted separately.

### Metagenomic sequencing and MAG analysis

Papillae biopsies were taken as described in Pacífico et al.^60^ from seven rumen cannulated Holstein cows at the start of a recent feeding trial^61^, before the animals were administered any specific diet. All procedures involving animal handling and treatment were approved by the institutional ethics committee of the University of Veterinary Medicine (Vetmeduni) Vienna and the national authority according to §26 of the Law for Animal Experiments, Tierversuchsgesetz 2012- TVG (GZ: BMWFW-68.205/0023-WF/V/3b/2015 and BMNWF- 68.205/0003-V/3b/2019). In the lab, biopsies were thawed on sterile microscope slides and the surface keratinous layer was scrapped off using a scalpel. This was done to enrich for bacterial DNA relative to the DNA from host epithelial cells. DNA extraction was then carried out using the PowerSoil Pro kit (Qiagen), paired-end libraries were prepared using the Westburg NGS DNA Library Prep Kit, and metagenomic sequencing was done on an Illumina Novoseq 6000 instrument with a 250 bp read length at the Vienna BioCenter Core facilities. The reads were trimmed using Trimmomatic (v. 0.39) (ref. 62) and mapped against a Bos Taurus reference genome (GCF_000003055.6) to filter out any reads obtained from the host. The remaining reads were assembled using SPAdes (v. 3.15.2) (ref. 63) and were mapped using BWA-MEM (v. 0.7.17) (ref. 64), before MAGs were generated using Metabat2 (v. 2.12.2) (ref. 63) with a minimum contig size of 1,500 bp. The MAGs were assessed for completeness and contamination using checkM (v. 1.1.3) (ref. 65), and then classified using the classify workflow from the genome taxonomy database toolkit (GTDB-Tk, v. 2.1.0) (ref. 65). Filtered reads were re-mapped to the MAGs using BWA-MEM (v. 0.7.17) (ref. 64) and the reads per kilobase (kB) were calculated in R (v. 3.6.3). The median reads per kilobase for the top ten most abundant MAGs, based on total reads per kilobase, with over 50% completeness and less than 10% contamination were then plotted in Fig. 1b.

The publicly available metatranscriptomes that were re-analysed were downloaded with fasterq-dump (v. 2.11.0) and mapped to the MAGs using BWA-MEM (v. 0.7.17) (ref. 64) (Extended Data Fig. 1). ORFs in the MAGs were then predicted using prokka (v. 1.14.6) (ref. 66) and reads mapping to predicted ORFs were counted using htseq-count (v. 0.11.3) (ref. 67). As above, the reads per kilobase were calculated in R (v. 3.6.3) and plotted for MAGs with over 50% completeness and less than 10% contamination.

### Cultivation approach and isolate screening

All strains were cultivated using a tryptic soy broth agar with the addition of 0.5 g l^−1^ l-cysteine HCl. The agar medium was always prepared and used on the same day. After autoclaving, 5 mM sodium nitrate, 5 mM sodium fumarate and 2.5 mM sodium formate were added to the medium. Further, 20 μg ml^−1^ nalidixic acid and 5 μg ml^−1^ vancomycin were added to select for the Campylobacteraceae over other rumen microbes, which was based on recent work to enrich for *Campylobacter ureolyticus*^68^. For bacterial isolation, papillae samples from four different animals, which were also sampled for the metagenomic sequencing (above), were thawed on ice, rinsed with sterile PBS and transferred to a 1.5 ml Eppendorf tube with a single 6.35 mm ceramic bead (MP Biomedical, 116540424-CF). The tubes were then shaken on a vortex at full speed for 10 min using a Qiagen Vortex adapter (13000-V1-24). Six serial dilutions (1/10) were made using 1× PBS and 15 μl of the resulting dilutions were spread on agar plates, which were incubated anaerobically in a 2.5 l anaerobic jar and atmosphere generation sachet (Thermo Scientific, R685025, Biomeriex, 96124). After a week of incubation at 39 °C in the dark, single colonies were picked and re-streaked three times to ensure purity. To identify Campylobacteriaceae among these colonies, scraped biomass was transferred into 20 μl of 10 g l^−1^ Chelex 100 (Bio-Rad, 1421253), which was boiled for 10 min on a hot plate. From this, 1 μl was added to a PCR reaction using primers that targeted the originally observed ASV (forward; 5′-GGAGGACAACAGTTAGAAATGAC-3′, reverse; 5′-CGTTAGATTTCACAAGAGACTTGAT-3′). Sequences were confirmed to be identical to the ASV of interest by Sanger sequencing.

### Genome sequencing and diversity analysis

With isolates from the papillae of four different animals, biomass was scraped from agar plates and DNA extraction was then carried out using the PowerSoil Pro kit. A total of 34 isolates were sequenced in two batches, the first containing 10 isolates. For the first batch, paired-end libraries were prepared using the Westburg NGS DNA Library Prep Kit, and genomic sequencing was carried out on a MiSeq instrument with a 300 bp read length at the Vienna BioCenter Core facilities. Reads were trimmed using Trimmomatic (v. 0.39) (ref. 64), assembled using SPAdes (v. 3.15.2) (ref. 62) and contigs smaller than 1 kB were removed using PRINSEQ-lite (v. 0.20.4) (ref. 69). For the second batch, paired-end libraries were prepared using the NEBNext FS II DNA Library Prep Kit, and genomic sequencing was carried out on an Illumina Novaseq 6000 instrument with a 100 bp read length at the Joint Microbiome Facility, Vienna. These reads were trimmed using cutadapt (v. 2.10) and assembled using SPAdes (v. 3.14.1) (ref. 63). Contigs shorter than 1 kB were removed using seqtk (v. 1.3). The genomes with >1% contamination were filtered using the mmgenome2 package (v. 2.1.2) (ref. 70). For the two populations that are focused on in this study, five and eight genomes were obtained for ‘*Ca*. C. stinkeris’ and ‘*Ca*. C. noahi’, respectively. In total, 34 genomes were sequenced and assembled.

To obtain complete genomes, DNA from three reference genomes (C. stinkeris NA3, C. noahi NE2 and VBCF_01 NA2) was also sequenced using the Oxford Nanopore platform. Libraries were prepared using the Nanopore Native Barcoding Genomic DNA by Ligation (EXP-NBD196, SQK-LSK109) protocol and sequenced on the MinION Mk1C instrument using a FLO-MIN106 flowcell. The resulting reads were basecalled using Guppy (v. 3.0.3+7e7b7d0, Oxford Nanopore Technologies), assembled using pomoxis (v. 0.3.1, Oxford Nanopore Technologies), polished using metadaka (v. 1.4.3, Oxford Nanopore Technologies) and finally co-assembled with Illumina data using SPAdes (v. 3.15.2) (ref. 63). The 16S rRNA gene was extracted from all genomes using prokka (v. 1.14.6) (ref. 66), and then aligned and visualized using MUSCLE (v. 5) (ref. 71) in Genious (v. 9.1.8, https://www.geneious.com). To assess genome wide diversity, MAGs and genomes were compared pairwise using fastANI (v. 1.33) (ref. 72). The resulting ANI values were hierarchically clustered using a complete linkage algorithm and plotted in R (v. 3.6.3). On the basis of the observed clustering, the 13 genomes clustering with the more abundant MAG (MAG 73) were aligned using progressiveMAUVE (v. 2015.02.05) (ref. 73) and the phylogenetic tree in Fig. 1c was constructed with the JC69 (ref. 73) model using phyML (v. 2.2.3) (ref. 74). The same alignment was also divided into 2.5 kB windows and a phylogenetic tree was calculated with iqtree^75^ with the model set to GTR + F + I + G4 for each window. The pairwise phylogenetic distance was then averaged within and between populations in R (v. 3.6.3) and plotted for Extended Data Fig. 6a.

For the concatenated marker tree shown in Extended Data Fig. 5, an alignment was generated using the genome taxonomy database toolkit (GTDB-Tk, v. 2.1.0) (ref. 65). The tree was then generated using iqtree^75^ with automated model selection^76^ (LG + F + I + G4). The 16S rRNA alignment was constructed as above using the 16S rRNA from each of the genomes in the concatenated marker tree and a 16S rRNA tree was calculated with iqtree^75^ with the model set to GTR + F + I + G4. In both cases, the trees were constructed with ultrafast bootstrapping set to 1,000 (ref. 77).

### Gene content and SNP-level diversity analysis

For the two complete reference genomes, ORFs were predicted and annotated using prokka (v. 1.14.6) (ref. 66). The resulting gene sequences were compared with all other genome assemblies using blastn (v. 2.10.1+) (ref. 78), and only alignments with both a percentage identity and percentage alignment of 70% were kept for classifying core, flexible and population specific genes in R (v. 3.6.3), as depicted in Fig. 2. Genes that aligned with all other genomes in the sister population with identical alignment length were used to calculate dN/dS^29^ using ape (v. 5.5) in R (v. 3.6.3), the distribution of which is plotted in Extended Data Fig. 6b. Further, the identity of the blast of the alignments between populations was used to generate the distribution in Extended Data Fig. 7a. Predicted amino acid sequences from prokka (v. 1.14.6) (ref. 66) were also compared with those predicted in the MAGs using blastp (v. 2.10.1+) (ref. 78). Then for Fig. 2d, the pglH sequences were aligned with MUSCLE (v. 5) (ref. 71), and the protein tree was constructed with the LG model^79^ using phyML (v. 2.2.3) (ref. 74).

For the SNP analysis in Fig. 3, metagenomes were first competitively mapped with BWA-MEM (v. 0.7.17) (ref. 64) to the complete genomes and a set of genomes representing those clustering with MAG 61. For the genomes clustering with MAG 61, a single representative genome was used for each of the clusters that was within 1% divergence. SNPs were called and filtered using bcftools (v. 1.12) (ref. 80) and VCFtools (v. 0.1.16) (ref. 81) and then counted in 1 kB windows in R (v. 3.6.3). The largest SNP-free region was in the C. stinkeris NA3, and this region was aligned with the corresponding region in C. noahi NE2 in Genious (v. 9.1.8, https://www.geneious.com) using MUSCLE (v. 5) (ref. 71) and the identity was calculated over 1 kB windows and plotted in R (v. 3.6.3). Finally, reads that competitively mapped to ‘*Ca*. C. stinkeris’ and ‘*Ca*. C. noahi’ were extracted using samtools (v. 1.9) (ref. 80) and aligned to each other using blastn (v. 2.10.1+) (ref. 78). Those reads that aligned across their entire length were used to generate the the distribution in Extended Data Fig. 7b.

### dPCR assay and papillae dissection

To quantify the two populations in vivo, papillae biopsies were taken as described in ref. 22 from five animals. After thawing on ice, three crypts and apex sections (Extended Data Fig. 8) were taken from each and placed in a 1.5 ml Eppendorf tube. To these, 200 μl of 10 g l^−1^ Chelex 100 (Bio-Rad, 1421253) was added the tubes were placed at 99 °C on a hot plate shaking at 900 rpm. The tubes were then spun down briefly, and 1 μl was sampled for dPCR, which was conducted with chips on the Stilla Naica Crystal Digital PCR System. The mastermix contained Stilla Naica multiplex PCR Mix and 10 μM of each primer and probe. A ‘*Ca*. C. stinkeris’ specific region was targeted with a fluorescein-containing probe (forward; 5′-TGGGCGCAATGCTATTAT G-3′, reverse; 5′-CATTTCACGCCTAAACATAAC C-3′, probe; 5′-/56-FAM/CTGGTTTTG/ZEN/GCATAGATAAAAGCGGAGA/3IABkFQ/−3′), while the ‘*Ca*. C. noahi’ specific region was targeted by a phosphoramidite-containing probe (forward; 5′- CAC AAC GAC CAT TGT AAC GAT AAT-3′, reverse; 5′-CCT ACA ACC AGC CAC AGT C-3′, probe; 5′-/5HEX/TG GTT TGA A/ZEN/A CTA AAT GGC GAG TTG CA/3IABkFQ/−3′). Probes were designed and provided by Integrated DNA Technologies. After droplet generation, the following protocol was used to amplify the population specific targets: 95 °C for 10 min, 45 cycles of 95 °C for 10 s and 62 °C for 40 s. A Silla Naica Prism 3 reader was the used to detect droplets, which were analysed by Crystal Miner software (v. 2.4.0.3) to export the copy numbers for the two targets based on the default settings.

### Comparative metatranscriptomics and aarC analysis

Transcriptomes were mapped using the same approach as described for the metagenomic mapping above. Reads mapping to ORFs predicted with prokka (v. 1.14.6) (ref. 66) were counted with htseq-count (v. 0.11.3) (ref. 67). To be able to compare the expression of genes across populations, we aligned ORFs with blastn (v. 2.10.1+) (ref. 78) and compared genes with over 80% alignment identity in terms of the number of mapped reads. To ensure that genes could be clearly distinguished from each other during the competitive mapping, those with over 97.5% similarity were not compared. We then carried out the statistical analysis of differential expression using using the R (v. 3.6.3) package DESeq2 (v. 1.26.0) (ref. 82), the results of which are plotted in Fig. 4a. To assess the diversity of the *aarC* genes, those predicted by prokka (v. 1.14.6) (ref. 66) were taken from the reference genomes, and aligned using MUSCLE (v. 5) (ref. 71) in Genious (v. 9.1.8, https://www.geneious.com). Gene trees shown in Fig. 4b were constructed with the JC69 (ref. 73) model using phyML (v. 2.2.3) (ref. 74).

### Growth and fitness assays

We compared the growth of representative strains on agar in the presence of acetate and propionate using dPCR as the strains did not grow on liquid media. This was true only for the ‘*Ca*. C. stinkeris’ and ‘*Ca*. C. noahi’ strains, as all others grew in the cultivation media in liquid form. We further reasoned that improvements in growth on solid media may be more representative of the in vivo growth conditions than liquid, as the bacteria are attached to the epithelial wall. Using the same agar medium as for cultivation, strains were streaked out and allowed to grow anaerobically for 1 week at 39 °C. On the day of the experiment, fresh tryptic soy broth agar medium with 0.5 g l^−1^ l-cysteine HCl was prepared. After autoclaving, the medium without any further supplementation was used as a control. To the media representing the two treatments, 5 mM sodium acetate or sodium propionate was added. With each of the three different media (control, acetate and propionate), 1 ml was added to the wells of a sterile 24-well cell culture plate. Biomass was then collected from the agar plates by scraping and resuspending it in 2 ml of freshly prepared peptone broth containing 0.5 g l^−1^ l-cysteine HCl. The optical density of the two suspensions was standardised to 0.075 at 570 nm, and an equal mixture of the two re-suspended strains was prepared for the co-culture experiments. Cell culture plates containing agar were inoculated with either 20 μl of a single strain or 40 μl of a co-culture mixture and then incubated at 39 °C anaerobically (as described above). After 48 h and 72 h in the case of the single strains and co-culture mixture, respectively, the cells were collected by cutting out each agar circle from a well with a scalpel and placing it in a 15 ml Falcon tube. To the Falcon tube, 2 ml of 10 g l^−1^ Chelex 100 (Bio-Rad, 1421253) was added, and the mixture was boiled at 100 °C in a water bath for 45 min. The samples were then diluted 1/5 in sterile, DNA-free water, before 2 μl were used for dPCR, as described above. Copy numbers for the acetate and propionate treatments were compared with the base medium for calculating the fold change and standard deviation in R (v. 3.6.3), which are shown in Fig. 5a.

### Population tracking in vivo

The DNA extracted by Neubauer et al.^23^ was used to monitor populations using the dPCR assay and method described above. This study tested the effects of feed additives using eight cows in a change-over design where two cows were assigned to the control group for each of the four experimental runs. Each experimental run consisted of two periods where a high-grain diet was fed, which induced changes in ruminal SCFA concentrations, and papillae samples were taken at three timepoints (one before and two after the high-grain periods). From 94 samples, 2 μl of the extracted DNA was added to the mastermix for dPCR. The resulting copy number data were merged with the rumen SCFA data measured in Neubauer et al.^23^, and Pearson correlations were calculated using the cor function in R, as depicted in Fig. 5b. Correlation tests were conducted the cor.test function in R (v. 3.6.3), and *P* values lower than 0.05 were considered significant.

### Statistics and reproducibility

The source of various sequence datasets that were integrated in this study, number of samples and animals is provided in Supplementary Table 1. For the datasets used in this study, no statistical method was used to pre-determine sample size. Further, no data were excluded from the analysis, and the details required for reproducing any of the bioinformatic processing or analysis can be found on GitHub (see ‘Code availability’).

### Reporting summary

Further information on research design is available in the Nature Portfolio Reporting Summary linked to this article.

## Supplementary information

Reporting Summary

Supplementary Tables Supplementary Tables 1–5.

## Data Availability

The publicly available data that we re-analysed here were generated by Wetzels et al.^22^, Neubauer et al.^23^, Mann et al.^13^ and Tan et al.^83^ Additionally, we re-analysed the amplicon datasets compiled in the meta-analysis by Anderson et al.^11^. A summary of these datasets with the corresponding accession numbers is available in Supplementary Table 1. The metagenomic and genomic sequencing data from the rumen papillae samples are available on NCBI under the accession number PRJNA886670. The Bos Taurus reference genome was downloaded from the NCBI RefSeq database (GCF_000003055.6).

## Code availability

Scripts with all the custom analysis and commands described above can be found at https://github.com/cameronstrachan/RumenCampylobacter2022.
