# Supplementary material for: Differential carbon utilization enables co-existence of recently speciated Campylobacteraceae in the cow rumen epithelial microbiome
Source: Nat Microbiol. 2023 Jan 12;8(2):309–20. doi: 10.1038/s41564-022-01300-y (PMC9894753; doi:10.1038/s41564-022-01300-y)
Supplement: Supplementary file 1 — Reporting Summary [file 41564_2022_1300_MOESM1_ESM.pdf]

nature portfolio

Corresponding author(s): Martin Polz

Last updated by author(s): Nov 21, 2022

Reporting Summary

Nature Portfolio wishes to improve the reproducibility of the work that we publish. This form provides structure for consistency and transparency in reporting. For further information on Nature Portfolio policies, see our Editorial Policies and the Editorial Policy Checklist.

Statistics

For all statistical analyses, confirm that the following items are present in the figure legend, table legend, main text, or Methods section.

n/a	Confirmed	
☐	☒	The exact sample size (*n*) for each experimental group/condition, given as a discrete number and unit of measurement
☐	☒	A statement on whether measurements were taken from distinct samples or whether the same sample was measured repeatedly
☐	☒	The statistical test(s) used AND whether they are one- or two-sided<br>*Only common tests should be described solely by name; describe more complex techniques in the Methods section.*
☐	☒	A description of all covariates tested
☐	☒	A description of any assumptions or corrections, such as tests of normality and adjustment for multiple comparisons
☐	☒	A full description of the statistical parameters including central tendency (e.g. means) or other basic estimates (e.g. regression coefficient) AND variation (e.g. standard deviation) or associated estimates of uncertainty (e.g. confidence intervals)
☐	☒	For null hypothesis testing, the test statistic (e.g. *F*, *t*, *r*) with confidence intervals, effect sizes, degrees of freedom and *P* value noted<br>*Give P values as exact values whenever suitable.*
☒	☐	For Bayesian analysis, information on the choice of priors and Markov chain Monte Carlo settings
☒	☐	For hierarchical and complex designs, identification of the appropriate level for tests and full reporting of outcomes
☐	☒	Estimates of effect sizes (e.g. Cohen's *d*, Pearson's *r*), indicating how they were calculated

Our web collection on statistics for biologists contains articles on many of the points above.

Software and code

Policy information about availability of computer code

Data collection	fastq-dump v.2.11.0<br>fasterq-dump v.2.11.0
Data analysis	Scripts with all the custom analysis and commands can be found at [https://github.com/cameronstrachan/RumenCampylobacter2022].<br><br>Open source software:<br><br>R (v.3.6.3)<br>qiime2 v. 2021.4.0<br>rRDP v. 1.20.0<br>Trimmomatic v. 0.39<br>cutadapt v. 2.10<br>SPAdes v. 3.15.2 and v. 3.14.1<br>BWA-MEM v 0.7.17<br>Metabat2 v. 2.12.2;<br>checkM v. 1.1.3<br>GTDB-Tk v. 2.1.0<br>PRINSEQ-lite v. 0.20.4<br>seqtk v. 1.3<br>mmgenome2 v. 2.1.2<br>Guppy v. 3.0.3

pomoxis v. 0.3.1
metadaka v. 1.4.3
prokka v. 1.14.6
MUSCLE v. 5
fastANI v. 1.33
progressiveMAUVE v. 2015.02.05
phyML v. 2.2.3
blastn v.2.10.1+
bcftools v.1.12
VCFtools v.0.1.16
htseq-count v.0.11.3
DESeq2 v.1.26.0
GTDB-Tk v. 2.1.0
htseq-count v. 0.11.3
iqtree v. 1.6.12
ape v.5.5

Commercial software:

Genious v.9.1.8
Crystal Miner software v. 2.4.0.3

For manuscripts utilizing custom algorithms or software that are central to the research but not yet described in published literature, software must be made available to editors and reviewers. We strongly encourage code deposition in a community repository (e.g. GitHub). See the Nature Portfolio guidelines for submitting code & software for further information.

Data

Policy information about availability of data

All manuscripts must include a data availability statement. This statement should provide the following information, where applicable:

- Accession codes, unique identifiers, or web links for publicly available datasets
- A description of any restrictions on data availability
- For clinical datasets or third party data, please ensure that the statement adheres to our policy

The publicly available data that we reanalysed here were generated by Wetzels et al., Neubauer et al., Mann et al., and Tan et al. Additionally, we re-analysed the amplicon datasets compiled in the meta-analysis by Anderson et al. A summary of these datasets with the corresponding accession numbers is available in Supplemental Table 1. The metagenomic and genomic sequencing data from the rumen papillae samples are available on NCBI under the accession number PRJNA886670. The Bos Taurus reference genome was downloaded from the NCBI RefSeq database (GCF_000003055.6).

Human research participants

Policy information about studies involving human research participants and Sex and Gender in Research.

Reporting on sex and gender	The research did not involve human research participants.
Population characteristics	The research did not involve human research participants.
Recruitment	The research did not involve human research participants.
Ethics oversight	The research did not involve human research participants.

Note that full information on the approval of the study protocol must also be provided in the manuscript.

Field-specific reporting

Please select the one below that is the best fit for your research. If you are not sure, read the appropriate sections before making your selection.

☐ Life sciences ☐ Behavioural & social sciences ☒ Ecological, evolutionary & environmental sciences

For a reference copy of the document with all sections, see nature.com/documents/nr-reporting-summary-flat.pdf

Ecological, evolutionary & environmental sciences study design

All studies must disclose on these points even when the disclosure is negative.

Study description	The study was focused on a specific group of bacteria that colonize the epithelial tissue of the cow rumen. Papillae samples that were used to sequence metagenomes and cultivate the bacteria of interest were collected during a previous feeding trial using dairy cows

	(Pacífico et al. (2022). These samples came from 6 different animals. The bacteria were further measured by digital PCR in papillae samples from a different past feeding trail involving the same animals (Neubauer et al. (2019)). In this case, the extracted DNA from this feeding trial was provided to us.
Research sample	Rumen biopsies were collected from 9 ruminally-cannulated non-lactating Holstein cows as described in Pacífico et al. (2022).
Sampling strategy	In the cultivation and sequence based analysis, 4-6 cows were used to ensure that the results were not specific to an individual animal. To track the bacteria by digital PCR, samples from 8 animals across 12 time points were used as per Neubauer et al. (2019).
Data collection	Sequencing data was collected by the Vienna BioCenter Core facilities or the Joint Microbiome Facility, Vienna. Cameron Strachan collected all microbiological data in the lab. Measurement of the short chain fatty acids in the rumen content is described in Neubauer et al. (2019).
Timing and spatial scale	The dates of collection for rumen biopsies are described in Neubauer et al. (2019) and Pacífico et al. (2021). The samples were collected from the ventral rumen wall and pappilae were cut, as close to the base as possible, approximately 20 cm below the opening of the cannula.
Data exclusions	No data was excluded.
Reproducibility	To ensure that the findings were not animal specific, the bacterial populations and metagenomes were sampled from multiple animals. Further, the agar based assays were repeated to ensure that the relative bacterial growth advantages given by acetate and propionate were reproducible.
Randomization	The design of the feeding trails is described in Neubauer et al. (2019) and Pacífico et al. (2021).
Blinding	Blinding is not relevant to this study as animals were not grouped.

Did the study involve field work? ☐ Yes ☒ No

Reporting for specific materials, systems and methods

We require information from authors about some types of materials, experimental systems and methods used in many studies. Here, indicate whether each material, system or method listed is relevant to your study. If you are not sure if a list item applies to your research, read the appropriate section before selecting a response.

Materials & experimental systems

n/a	Involved in the study
☒	☐ Antibodies
☒	☐ Eukaryotic cell lines
☒	☐ Palaeontology and archaeology
☐	☒ Animals and other organisms
☒	☐ Clinical data
☒	☐ Dual use research of concern

Methods

n/a	Involved in the study
☒	☐ ChIP-seq
☒	☐ Flow cytometry
☒	☐ MRI-based neuroimaging

Animals and other research organisms

Policy information about studies involving animals; ARRIVE guidelines recommended for reporting animal research, and Sex and Gender in Research

Laboratory animals	The study did not involve laboratory animals.
Wild animals	The study did not involve wild animals.
Reporting on sex	The work here used samples taken from dairy cattle during previous feeding trials.
Field-collected samples	Sample collection and storage is described in Neubauer et al. (2019) and Pacífico et al. (2022). For metagenomic sequencing, the papillae were thawed at room temperature and the surface layer was scraped off for DNA extraction. In terms of cultivation, papillae were thawed on ice, rinsed with sterile PBS and then bead beaten. Details are provided in the methods section of the paper.
Ethics oversight	All procedures involving animal handling and treatment were approved by the institutional ethics committee of the University of Veterinary Medicine (Vetmeduni) Vienna and the national authority according to §26 of the Law for Animal Experiments, Tierversuchsgesetz 2012- TVG (GZ: BMWFW-68.205/0023-WF/V/3b/2015 and BMNWF- 68.205/0003-V/3b/2019).

Note that full information on the approval of the study protocol must also be provided in the manuscript.
